# Mouse Health Span: Why Lifespan is No Longer Enough

**DOI:** 10.3402/pba.v2i0.20276

**Published:** 2012-12-31

*Felipe Sierra, PhD* *Rochelle Buffenstein, PhD* *Steve Austad, PhD* *Arlan Richardson, PhD* Conference organizers

