# Supplementary material for: Mouse Health Span: Why Lifespan is No Longer Enough
Source: Pathobiol Aging Age Relat Dis. 2012 Dec 31;2:10.3402/pba.v2i0.20276. doi: 10.3402/pba.v2i0.20276 (PMC3535693; doi:10.3402/pba.v2i0.20276)
Supplement: Related texts

# Sub article: Mouse Health Span: Why Lifespan is No Longer Enough

**Published:** 2012-12-31

**Authors:** Felipe Sierra, Rochelle Buffenstein, Steve Austad, Arlan Richardson

## Abstract

The San Antonio Nathan Shock Center Conferences have attracted international speakers and participants since 1995. This annual conference, held in Bandera, Texas, addresses a different topic in the biology of aging each year. The venue's intimate setting, relatively remote location and common areas are ideal for a small conference (80–100 participants) where informal intellectual interchange supplements that of the formal sessions. The 2012 meeting, part of an annual series sponsored by the Nathan Shock Center of Excellence in the Biology of Aging and the Barshop Institute for Longevity and Aging Studies at the University of Texas Health Science Center San Antonio, addressed the concept that healthy aging and assessment of physiological performance are important parameters, in addition to longevity, to measure quality of life with increasing age.

The purpose of the 2012 conference was to provide a forum for the presentation and discussion of various assays of measuring physiological performance and function and determining what assays of function could be used to asses healthspan of a mouse. Longevity is a precise endpoint (binary, the individual is either alive or it is dead), but the true goal of aging research is to increase the health of the elderly, not their longevity. That is, the goal is to enhance and extend healthspan, defined as the portion of our lives spent free of serious illnesses and disabilities. The assumption is that the only way an organism can increase its lifespan is by increasing its healthspan. This is a plausible assumption, but it still needs to be proven each time a manipulation is assessed for its potential for translation into humans. While the invertebrate models are particularly useful in genetic studies, they are generally not very good models for mammalian health, physiology, disease susceptibility, etc. Mice age with a constellation of diseases and functional losses that in some aspects resemble those observed in humans. Therefore, the conference focused on healthspan measures in mice. To this end, speakers were recruited who are working on assays (both simple and complex) to evaluate the functional status of various organ and physiological systems that are important in the health/physiological performance in mice and/or humans. In addition, attention was given to clarification of the molecular mechanisms underlying physiological decline, and its causal relationship to metabolic changes, muscle wasting, neurodegenerative diseases, cardiovascular disease, cancer, and inflammation and immunity, as well as targets for prophylactic intervention. Thus, the conference gave investigators a panel of assays that would allow them to determine the effect of genetic or pharmacological/nutritional manipulation on healthspan.

Abstracts from posters presented at the meeting are presented in this special abstract issue to provide an overview of the breadth and depth of the program.

# Sub article: Chronic rapamycin restores vascular integrity and improves memory after the onset of Alzheimer's-like disease in mice

**Published:** 2012-12-31

**Authors:** Jonathan J. Halloran, Ai-Ling Lin, Wei Zheng, Raquel R. Burbank, Stacy A. Hussong, Natalia Podlutskaya, Matthew J. Hart, Martin Javors, Randy Strong, Arlan G. Richardson, James D. Lechleiter, Peter T. Fox, Veronica Galvan

**Affiliations:** 1 Department of Physiology; 2 The Barshop Institute for Longevity and Aging Studies; 3 Research Imaging Institute; 4 Department of Cellular and Structural Biology; 5 Department of Molecular Medicine; 6 Department of Pharmacology; 7 Department of Psychiatry, University of Texas Health Science Center at San Antonio; 8 Geriatric Research, Education and Clinical Center and Research Service, South Texas Veterans Health Care System, San Antonio, TX, USA

## Abstract

Vascular pathology is a major feature of Alzheimer's disease (AD) and other dementias. We recently showed that chronic administration of the target-of-rapamycin (TOR) inhibitor rapamycin, a drug that extends lifespan and delays aging, prevented the development of AD-like disease in mice modeling AD. Here we show that rapamycin administrated after the onset of AD-like deficits reversed brain vascular breakdown through endothelial nitric oxide (NO) synthase activation and NO-dependent vasodilation, decreased cerebral amyloid angiopathy and brain microhemorrhages, and improved memory in AD mice. These data suggest a mechanism by which chronic rapamycin ameliorates established AD-like deficits through the preservation of brain vascular integrity and function. Rapamycin, an FDA-approved drug already used in the clinic, may have promise as a therapy for AD and possibly for vascular dementias.

# Sub article: Caloric restriction-induced preserved brain metabolism in aged rats

**Published:** 2012-12-31

**Authors:** Ai-Ling Lin, Daniel Coman, Douglas Rothman, Fahmeed Hyder

**Affiliations:** 1 Research Imaging institute; 2 Barshop Institute for Longevity and Aging Studies; 3 Department of Cellular and Structural Biology, University of Texas Health Science Center, San Antonio, TX, USA; 4 Magnetic Resonance Research Center, Yale University, New Haven, CT, USA

## Abstract

A widely accepted cause of the functional losses that accompany aging is decreased brain metabolism (i.e., glucose oxidative capacity in mitochondria). It is generally believed that preserving bioenergetics is critical for optimizing lifespan and healthspan. Interventions have been introduced to preserve metabolism in aging process. Caloric restriction (CR) perhaps is the most well-studied one for various model organisms of extended longevity. In addition, in the neuronal system of rats (F344BNF1), CR also enhances cognitive function. However, the underlying physiology in the brain remains unclear. In the study, we used carbon-13 magnetic resonance spectroscopy (C-13 MRS) to investigate CR effect on brain metabolism in aged rats (24 months of age). CR-treated and control F344BNF1 rats (N = 6 for each group) were purchased from NIA. C-13 labeled glucose was continuously infused through the femoral vein of the rat for two hours and MRS was acquired simultaneously. The results show that CR rats had significantly increased oxidative metabolism rate (Voxi) in neurons (p < 0.01) and neurotransmission rate (glutamate-glutamine recycling rate; Vcyc) (p < 0.01) compared to the controls. The aged CR rats’ Voxi (4.5 µmol/g/min) and Vcyc (2.2 µmol/g/min) were comparable to those of young control rats reported in literature. However, CR and control rats did not have significant difference of glucose uptake and lactate production in the brain. The results suggest that alternative fuel subtract (e.g., ketone bodies) may be used to meet the brain energy demand. Our data provide a possible explanation of CR-induced increased lifespan and healthspan in rats.

# Sub article: Does rapamycin protect the hippocampal formation from a loss of inhibitory control?

**Published:** 2012-12-31

**Authors:** Sunayana Korde, Veronica Galvan, David Jaffe

**Affiliations:** 1 Department of Biology and The Neurosciences Institute, University of Texas at San Antonio; 2 Department of Physiology and Barshop Institute, UTHSCSA, San Antonio, TX, USA

## Abstract

Chronic administration of rapamycin by transgenic (Tg) PDAPP mice allows them to perform better in hippocampal-associated learning and memory tasks compared with controls. We found, using conventional brain slice methods, that rapamycin had no significant effect on excitatory synaptic transmission, neuronal excitability, or the induction of long-term potentiation (LTP) in the CA1 region of the hippocampus. Surprisingly, we observed no significant effect on LTP in the control Tg group compare with wild type (Wt). We were concerned that some factor, such as stress due to transportation, might have enhanced the likelihood for LTP. To test for this possibility, we examined the relationship between stimulus strength and the magnitude of LTP induction. It is well known that LTP is a function of stimulus strength before induction due to the properties of NMDA receptors; with greater depolarization there is more calcium influx and, in turn, larger LTP. However, we found no correlation for either of our non-rapa control groups (Wt or Tg). In contrast, there was a correlation when animals were administered rapamycin, and the correlation was greater for Wt over Tg animals. Our working hypothesis is that stress, possibly due to transport, depressed inhibitory circuits lowering the threshold for LTP induction. Monte Carlo simulations comparing the amount of LTP produced by variations in the ratio of excitation to inhibition (E/I) support this hypothesis. Chronic rapamycin may protect the hippocampal network from dis-inhibition, maintaining E/I to sustain normal cognitive function.

# Sub article: Vision health span vs. lifespan: Rapamycin treatment does not prevent age-related decline in an optokinetic visual behavior or in retinal neuron number

**Published:** 2012-12-31

**Authors:** Rene Carlos Rentería, A. Vasalauskaite, N.P. Akimov

**Affiliations:** 1 Department of Physiology; 2 Center for Biomedical Neuroscience, University of Texas Health Science Center at San Antonio, San Antonio, TX, USA

## Abstract

Chronic treatment with the mTOR inhibitor rapamycin (“Rapa”) extends lifespan in mice. Whether Rapa slows specific aging processes to increase “health span” is unknown. During aging, visual performance declines, and retinal neurons decrease in number. Here, we find and quantify a specific age-related decline in vision in mice. We also show that Rapa does not prevent this decline in visual function or affect neuron number. Instead, Rapa was detrimental to vision. Vision was tested using optokinetic tracking (“OKT”) to measure spatial frequency threshold at maximum contrast (“SPFT”) of the head-tracking behavior to horizontally drifting sinusoidal gratings. Male B6 mice were tested at 5, 21, 29, and 33 months of age (“mos”). Two other lines of mice were fed chow ad libitum containing micro-encapsulated rapamycin from 3 until 18 mos. In another group of B6 mice treated with rapamycin from 4 to 25 mos, retinas were immunostained with markers to count neuron subtypes. During normal aging, OKT SPFT significantly declined by 31%. Rapa did not protect against this age-related OKT decline in either treated strain but significantly decreased OKT performance for male, but not female, mice at 18 mos. Rapa male mice had decreased IPL thickness in the retinal periphery, but numbers of dopaminergic and cholinergic amacrine neurons and retinal ganglion cells were unchanged. Thus, Rapa does not prevent age-related declines in OKT visual function or in retinal neuron number. It instead causes an OKT SPFT deficit in male mice. These findings suggest Rapa does not increase vision health span during aging.

# Sub article: Novel olfactory acuity assay for assessment of health span in mice

**Published:** 2012-12-31

**Authors:** Samantha Rendón, Kathleen E. Fischer, Steven N. Austad

**Affiliations:** 1 Barshop Institute for Longevity and Aging Studies; 2 Department of Cellular & Structural Biology, University of Texas Health Science Center San Antonio; 3 Department of Physiology, University of Texas Health Science Center, San Antonio, TX, USA

## Abstract

Decline in sensory acuity is a general hallmark of aging, which in humans decreases quality of life. We report here creation and successful utilization of a novel sensory acuity assay in mice. Three features of the assay merit attention. First, as mice are primarily nocturnal in nature, olfaction is an important sensory modality for them. Second, our assay instead of using artificial olfactory cues employs major urinary proteins, which are important in both intrasexual and intersexual communication of mice in nature. Third, the assay can be performed in the mouse's home cage, thus avoiding artifacts from distracting, novel environments. Procedurally, the assay uses serial dilutions of urine and preference for the urinary odor relative to a water control to measure olfactory acuity. Age-related changes in olfactory acuity have not previously been reported in mice. We created this assay, which compares time spent sniffing a sample relative to time spent at a distilled water control. It has been used numerous times and proves to be sensitive, repeatable and encompass particularly informative urinary dilution ranges. Specifically, previous testing revealed that of any age, mice usually cannot distinguish urine from water at a dilution of 1:10,000 (Rendón, unpublished data). The range of experimental dilutions between 1:10,000 to 1:5,000 has been narrowed down through successive modifications. Sampling in this range, we have detected a clearly defined age-related decline in mouse olfactory acuity. Therefore, this assay will serve useful in assessing changes in health span of mice and can be combined with therapeutic agents to assist in evaluation of their effect on health span.

# Sub article: Protein oxidation may be an important regulator in the development of insulin resistance

**Published:** 2012-12-31

**Authors:** Jennalynn Styskal, Adam Salmon, Nicholas Musi, Arlan Richardson

**Affiliations:** 1 Department of Cellular and Structural Biology; 2 Department of Molecular Medicine; 3 Department of Medicine; 4 Barshop Institute for Longevity and Aging Studies, University of Texas Health Science Center at, San Antonio, TX, USA; 5 Geriatric Research, Education and Research Center, South Texas Veterans Health Care System, San Antonio, TX, USA

## Abstract

The accumulation of oxidative damage is a proposed mechanism regulating the aging process and the development of disease. Proteins are sensitive to such oxidative stress, which can cause them to accumulate, altering conformational structure, and thus the function, of cellular proteins. Methionine sulfoxide reductase A (MsrA) plays an important role in the antioxidant defense, but is unique in that it repairs protein oxidative damage. MsrA reduces methionine sulfoxide residues to non-oxidized methionine, thus participating in the antioxidant defense system of cells specifically by protecting proteins from oxidative stress. We have found that mice that lack MsrA (MsrA^−/−^) and mice that over express MsrA (MsrA^Tg^) are phenotypically similar to wildtype (WT) mice under normal conditions, but that MsrA levels can regulate susceptibility to oxidative stress. Because these mice are grossly normal, this suggests that excess methionine oxidation may not occur under these physiological conditions. In vivo, increasing adiposity has been associated with increases in oxidative stress, altered redox signaling and increased oxidative damage to cellular macromolecules in several disease models, including obesity-induced metabolic diseases. When placed on a high fat (HF) diet, MsrA^−/−^ mice become more insulin resistant than WT mice whereas MsrA^Tg^ mice are protected from development of insulin resistance. The increase in insulin resistance in MsrA^−/−^ mice fed HF diets correlated with reduced insulin-stimulated signaling in the insulin signaling pathway. We found that HF fed MsrA^−/−^ mice had reduced phosphorylation of both insulin receptor and Akt with administration of insulin under high fat fed conditions. Also, increased insulin sensitivity seen in the HF fed MsrA^Tg^ mice correlated with an increase in insulin-stimulated signaling in the insulin signaling pathway. These results suggest that oxidative damage, specifically to proteins, may play an important role in obesity-induced insulin resistance. To address how protein oxidation may cause insulin resistance, we have utilized in vitro studies in primary myoblasts to test the effect of MsrA on oxidative stress-induced insulin resistance. By utilizing these models, this study will test the hypothesis that MsrA can regulate the development of insulin resistance by repairing oxidative damage in proteins involved in the insulin signaling pathway in vitro. Insulin resistance can be induced in vitro by H_2_O_2_. In this study, skeletal muscle precursor cells isolated from MsrA^−/−^, MsrA^TG^, and WT mice, and then differentiated into myotubes, were tested for resistance to oxidative stress. Insulin signaling protein phosphorylation correlates with in vivo signaling observations, determined by western blot after insulin stimulation. Our hypothesis is that the level of protein oxidation can be correlated with the degree of insulin resistance in this system. Protein oxidation can be globally measured in the cell using a carbonyl assay. Once labeled, individual proteins can also be measured for total carbonyl content via immune precipitation. Because oxidation of proteins can lead to a decline in their function, these studies will focus on both function of the insulin signaling proteins isolated from these models as well as oxidation status of these proteins.

# Sub article: Maintaining mitochondrial energetics through long-term and acute modification of mitochondrial oxidative stress

**Published:** 2012-12-31

**Authors:** Shane Kruse, Michael P. Siegel, Hazel H. Szeto, David J. Marcinek

**Affiliations:** 1 University of Washington, Department of Radiology, Seattle, WA, USA; 2 University of Washington, Department of Bioengineering, Seattle, WA, USA; 3 Weill Cornell Medical College, New York, NY, USA

## Abstract

Loss of skeletal muscle function is severely debilitating and sarcopenia profoundly affects the quality of life in the aged population. Impaired mitochondrial energetics in skeletal muscle is associated with loss of function and increased mitochondrial oxidative stress. To explore age-related mitochondrial energy deficits we use chronic (transgenic) and acute (pharmacological) targeting of mitochondrial oxidative stress. Previous work demonstrated that mitochondrial targeted catalase (mCAT) delays the onset of age-related pathology and extends lifespan in mice. However, little is known about how the relationship of mitochondrial energetics and cellular redox status changes with age. We demonstrate that there is a decline in mitochondrial quality in aged permeabilized skeletal muscle, particularly in the fast-twitch extensor digitorum longus, that was prevented in mice expressing mCAT. We also demonstrate that acute treatment (~1hr) of aged mice with the mitochondria-targeted small peptide SS-31 results in immediate improvement of skeletal muscle energy metabolism and performance. These results provide further evidence that decreased mitochondrial function with age may be due to an altered oxidative status of mitochondria and we propose that there are two facets of mitochondrial deterioration with age: a structural component that is attenuated with long-term expression of MCAT, and a regulatory component dependent upon the oxidative status of the cell that is rapidly reversible with acute treatment of SS-31. These results suggest that the oxidative state of skeletal muscle is a practical therapeutic target, and raises questions about how oxidative status and mitochondrial content affect the adaptive and pathological response of mitochondrial metabolism to age.

# Sub article: Effects of Cu/ZnSOD overexpression on aging under obese and non-obese conditions in rats

**Published:** 2012-12-31

**Authors:** Sara Dube, L.C. Flores, A.B. Salmon, M. Ortiz, M. Roman, N. Musi, W. Qi, S. Lee, G.B. Hubbard, Holly Van Remmen, A. Bhattacharya, Y. Liu, J. Kirkland, T. Pirtskhalava, T. Tchkonia, Arlan Richardson, Y. Ikeno

**Affiliations:** 1 Barshop Institute for Longevity and Aging Studies; 2 Department of Pathology; 3 Cellular and Structural Biology; 4 Molecular Medicine; 5 Medicine, University of Texas Health Science Center at San Antonio; 6 Research Service, GRECC, Audie Murphy VA Hospital, STVHCS, San Antonio, TX, USA; 7 Robert and Arlene Kogod Center on Aging, Mayo Clinic

## Abstract

Recently, our laboratory made the surprising observation that overexpressing Cu/ZnSOD [Tg(SOD1-SD)^+/0^] in Sprague-Dawley (SD) rats resulted in a significant increase in lifespan and a reduction in age-related pathologies. The purpose of this study was to determine why overexpressing Cu/ZnSOD increases lifespan in SD rats. The Tg(SOD1-SD)^+/0^ rats showed lower levels of oxidative damage to DNA and lipids in vivo and higher resistance to oxidative stress in vitro. Both Tg(SOD1-SD)^+/0^ and wild-type rats showed an age-related increase in body fat and Cu/ZnSOD overexpression did not attenuate adiposity. Interestingly, Tg(SOD1-SD)^+/0^ rats showed a significant increase in insulin sensitivity at a young age and lower plasma glucose levels at an old age. To further investigate the role of Cu/ZnSOD overexpression on aging, we generated transgenic rats with F344 overexpressing Cu/ZnSOD [Tg(SOD1-F344)^+/0^]. Tg(SOD1-F344)^+/0^ rats showed similar levels of Cu/ZnSOD overexpression to Tg(SOD1-SD)^+/0^. The Tg(SOD1-F344)^+/0^ rats showed lower levels of oxidative damage to lipids in vivo, however, neither Tg(SOD1-F344)^+/0^ nor wild-type rats showed any age-related changes in body fat, insulin insensitivity, and plasma glucose levels. Furthermore, Tg(SOD1-F344)^+/0^ rats showed little increase in lifespan compared to wild-type rats. Our results are very exciting because these data indicate that overexpression of Cu/ZnSOD could be more protective against oxidative stress and could attenuate aging and age-related diseases under obese conditions in mammals. (Supported by grants from the VA Merit Review, the American Federation for Aging Research, and the Glenn Foundation)

# Sub article: Oxidative damage promotes a decline in the regulation of glucose metabolism

**Published:** 2012-12-31

**Authors:** Adam B. Salmon, Jenna Lynn Styskal, Arlan Richardson

**Affiliations:** 1 The Geriatric Research Education and Clinical Center, South Texas Veterans Health Care System, San Antonio, TX, USA; 2 The Sam and Ann Barshop Institute for Longevity and Aging Studies, The University of Texas Health Science Center at San Antonio, San Antonio, TX, USA

## Abstract

A reduced ability to effectively regulate glucose metabolism is one of the most common markers of declining healthspan in aging mammals. Advancing age is an independent risk factor in the development of glucose intolerance, insulin resistance, and diabetes mellitus. Understanding the mechanisms responsible for this could significantly contribute to developing effective therapeutics or preventatives for those most at risk. Our data support a hypothesis that oxidation of proteins involved in insulin signaling may play a significant role in this process. Using a cell culture model, we show that oxidative stress inhibits the cellular response to insulin. The binding of insulin to insulin receptor normally promotes auto-phosphorylation of the β-subunit which regulates downstream insulin signaling through its kinase activity. Our data show that oxidative stress inhibits insulin signaling partly by causing oxidative damage that inhibits this process. Oxidative stress promotes formation of protein carbonyl adducts within insulin receptor; these adducts lead to diminished auto-phosphorylation function. We then addressed whether insulin receptor oxidation occurs in vivo with metabolic dysfunction. Insulin receptor isolated from high fat-fed C57BL/6 mice also show significantly elevated insulin receptor oxidative damage and reduced auto-phosphorylation function. Our preliminary studies suggest a similar process of oxidative damage is associated with reduced glucose metabolism in aging mice. These data support the idea that accumulating oxidative damage is a common molecular mechanism by which several primary risk factors (i.e., obesity, aging) promote insulin resistance. Targeting therapeutics that reduce/remove/repair oxidative damage might then develop as a valuable treatment option among the geriatric population.

# Sub article: The dietary effects of high-fat/high-sucrose feeding in female PAPP-A (-/-) mice

**Published:** 2012-12-31

**Authors:** Cristal M. Hill, O. Arum, F. Wang, R. Boparai, Y. Fang, A. Spong, R. Westbrook, M.M. Masternak, A. Bartke

**Affiliations:** 1 Department of Medical Microbiology, Immunology, and Cell Biology; 2 Department of Internal Medicine-Division of Geriatric Research; 3 Department of Physiology, Southern Illinois University-School of Medicine; 4 Burnett School of Biomedical Sciences, University of Central Florida, FL, USA

## Abstract

Longevity and aging are influenced by common intracellular signals of the insulin and IGF-1 pathway. Enhanced availability of IGF-1 is promoted by cleavage of IGF binding proteins (IGFBPs) by proteases, including the pregnancy-associated plasma protein-A (PAPP-A). PAPP-A (-/-) mice live 30% longer than their normal littermates and have decreased bioactive IGF-1 on normal diets. Our objective was to elucidate the effects of a high-fat (58 % kcal)/ high-sucrose (25.5 % kcal) diet that promotes obesity and increase pro-inflammatory cytokines in normal and PAPP-A(-/-) female littermates. The results indicate that PAPP-A (-/-) mice fed a high energy diet are more glucose tolerant than normal littermates fed a low energy diet (P ≤ 0.05) while insulin tolerance did not change. The high energy diet increased IGF-1 levels in PAPP-A (-/-) mice compared to littermates (-/-) fed a low energy diet (P ≤ 0.002). PAPP-A (-/-) mice fed with a high energy diet had lower levels of pro-inflammatory cytokines (IL-2, IL-6 and TNF-a) compared to normal littermates fed a high energy diet (P < 0.05). In contrast, anti-inflammatory cytokine levels (IL-4 and adiponectin) were higher in PAPP-A (-/-) mice fed a high energy diet compared to normal littermates on high energy diet (P < 0.05). We conclude that PAPP-A (-/-) mice when compared to normal littermates are resistant to the effects of diet-induced metabolic dysfunction. Furthermore, high energy fed PAPP-A (-/-) mice have higher levels of anti-inflammatory cytokines and lower levels of inflammatory cytokines, possibly rescuing them from the detrimental effects of a high energy diet.

# Sub article: Rapamycin increases adipose tissue mitochondrial biogenesis and fatty acid oxidation in a mouse model of type 2 diabetes

**Published:** 2012-12-31

**Authors:** Deepa Sathyaseelan, Michael Walsh, Ryan Hamilton, Daniel Pulliam, Yun Shi, Holly Van Remmen

**Affiliations:** 1 The Barshop Institute of Longevity and Aging Research; 2 Department of Cellular and Structural Biology, The University of Texas Health Science Center at San Antonio, TX, USA; 3 Geriatric Research, Education and Clinical Center, South Texas Veterans Health Care System, San Antonio, TX, USA

## Abstract

Obesity is a major risk factor for the development of age-related metabolic diseases. The mammalian target of the rapamycin (mTOR) pathway plays critical roles in eukaryotic cell growth, survival, and translation and hyperactivation of mTOR pathway due to excess nutrients causes insulin resistance, a major risk factor for type 2 diabetes. Rapamycin is a potent inhibitor of mTOR pathway suggesting its beneficial effects on metabolism. Paradoxically, rapamycin treatment causes glucose intolerance in mice. While most of the studies focus on the effect of rapamycin on metabolism in normal mice, no study has addressed the metabolic effects of rapamycin in diabetic mouse models. Here, we are studying the effects of rapamycin in db/db mice, a model of diabetic dyslipidemia. Administration of rapamycin for 9 months, starting at 2 months of age, significantly reduced body weight (43%) in female db/db mice compared to db/db mice fed the control diet (eudragit), due to a reduction in fat mass. This reduction in fat mass is not due to alterations in fat synthesis (PPARξ and SREBP1) or fatty acid transport (CD36 and FATP1) or lipolysis (P-HSL/HSL ratio), rather due to increased levels fatty acid oxidation as indicated by increased levels of carnitine palmitoyltransferase I (CPT1, 5-folds), large-chain acyl-coenzyme A dehydrogenase (LCAD, 2.5-folds) and medium-chain acyl-coenzyme A dehydrogenase (MCAD, 1.5-folds) in rapamycin-fed db/db mice compared to eudragit-fed db/db mice. Consistent with this observation, peroxisome proliferator-activated receptor gamma coactivator 1-alpha (PGC1-α) are significantly up-regulated both at the transcriptional and translational levels. In addition, markers of mitochondrial biogenesis CREB-regulated transcription coactivator 3 (CRTC3), nuclear respiratory factor 1 (NRF1) and estrogen-related receptor alpha (ERRα) were significantly elevated in the adipose tissue of rapamycin-fed db/db mice. While rapamycin did not decrease the levels of circulating triglycerides and glucose in db/db mice, levels of circulating free fatty acid was significantly reduced and adiponectin levels were significantly increased by rapamycin treatment, suggesting improved insulin sensitivity. Finally, insulin sensitivity assessed by insulin tolerance test showed significant improvement in insulin sensitivity in rapamycin-fed db/db mice. In summary, our study demonstrates for the first time that rapamycin exhibits anti-obesity effect in db/db mice and improves insulin sensitivity due to the up-regulation of the mitochondrial biogenesis and increased fatty acid oxidation in the white adipose tissue.

# Sub article: A mutation in a complex IV assembly protein (sco2) leads to significant decreases in complex IV activity and distinctive metabolic phenotypes

**Published:** 2012-12-31

**Authors:** Shauna Hill, Daniel Pulliam, Deepa Sathyaseelan, Yuhong Liu, Holly Van Remmen

**Affiliations:** 1 Department of Cellular and Structural Biology, University of Texas Health Science Center at San Antonio, San Antonio, TX, USA; 2 Barshop Institute for Longevity and Aging Studies, University of Texas Health Science Center at San Antonio, San Antonio, TX, USA; 3 GRECC, South Texas Veterans Health Care System, San Antonio, TX, USA

## Abstract

Cytochrome c oxidase (COX) is an essential transmembrane protein complex in the mitochondrial respiratory electron chain. Mutations in genes responsible for the assembly of COX are associated with Leigh syndrome, cardiomyopathy, spinal muscular atrophy and other fatal metabolic disorders in humans. Paradoxically, mice lacking the COX assembly protein SURF1 show increased longevity associated with upregulation of mitochondrial biogenesis and stress response pathways despite significant reductions in COX activity. Here we asked whether a mouse model of cytochrome c oxidase deficiency due to a mutation in the sco2 gene, a copper chaperone that is required for the activity of COX would have similar molecular and physiologic changes. A complete knockout of the sco2 gene in mice is embryonic lethal, however mice harboring a mutated sco2 knock-in (KI) allele that is commonly found in human patients with sco2 mutations is viable, and despite the 30–60% reduction in COX activity, no significant phenotypic abnormalities are readily apparent. Interestingly, these mice have a decrease in lean mass and increase in fat mass. Preliminary evidence suggests that these mice are insulin resistant and glucose intolerant as compared to wild-type mice. The sco2 KI/KI mice also have decreased running endurance on the treadmill suggesting that these mice have muscle weakness. Interestingly, the COX-deficient mice do not have changes in the blood lactate levels suggesting that these mice do not upregulate glycolysis to compensate for decreased rates of respiration. This is counter to studies done in another COX deficient Surf1-/- mice, illuminating the complex nature of mitochondrial dysfunction on physiology. Results from this study will further our understanding of the role of complex IV in physiological outcomes due to mitochondrial dysfunction.

# Sub article: Physical performance in an animal model of Vitamin D insufficiency

**Published:** 2012-12-31

**Authors:** Kim L. Seldeen, Martin Pang, M. Rodríguez-Gonzalez, M. Hernandez, P. Yu, Bruce R. Troen

**Affiliations:** Division of Gerontology and Geriatric Medicine, University of Miami Miller School of Medicine, Miami VA Geriatric Research Education and Clinical Center, Miami, FL, USA

## Abstract

Vitamin D insufficiency, sarcopenia of aging, and obesity exert profound impacts on physical performance and overall healthspan. Although human clinical studies have demonstrated significant relationships between vitamin D and physical performance, they contain confounding factors such as age, obesity, diet, and lifestyle that make understanding the specific pathophysiology difficult. Therefore, we are developing a novel mouse model capable of isolating individual and combinatorial impacts of vitamin D insufficiency, aging and obesity on physical performance. We provided 6 month-old male mice with either 1000IU or 125IU vitamin D_3_/kg chow over 4 months. Longitudinal serum 25-OH vitamin D measurements show levels change rapidly (both depletion and repletion) and consistently to the degree of supplementation, allowing for comparisons between sufficient and insufficient mice. Furthermore, our data indicate body weight and fat percentage are higher in vitamin D insufficient mice after 4 months. Additionally, our data suggest that vitamin D insufficient mice have higher levels of IL-6 and TNF- in their epididymal fat tissue. Rotarod treadmill performance and grip strength were similar regardless of vitamin D status. However, we found that elderly mice (24 months) exhibit functional decline compared to young mice despite both groups being sufficient (25-OHD ≥ 30 ng/ml). These data lay the foundation for our continuing investigation on vitamin D insufficiency, aging, obesity and physical performance and will further our understanding of the underlying mechanisms driving health span decline.

# Sub article: Rapamycin attenuates motor deficit in a mouse model of synucleinopathies

**Published:** 2012-12-31

**Authors:** Xiang Bai, Margaret Chia-Ying Wey, Anthony Martinez, Vanessa Martinez, Elizabeth Fernandez, Randy Strong

**Affiliations:** 1 The Barshop Institute for Longevity and Aging Studies; 2 Department of Pharmacology, University of Texas Health Science Center at San Antonio, TX, USA; 3 Geriatric Research, Education and Clinical Center and Research Service, South Texas Veterans Health Care System, San Antonio, TX, USA

## Abstract

Synucleinopathies are age-related neurodegenerative disorders characterized by expression of pathological α-synuclein inclusions. Synucleinopathies include Parkinson's disease (PD), multiple system atrophy and dementia with Lewy bodies (DLB), which affect millions of patients worldwide. Parkinsonian motor symptoms like rigidity and slow movement are common in synucleinopathies. A53T mutation is the first α-synuclein mutation linked to PD, and it is linked to both sporadic and familial PD. Autophagy is reduced in PD brain. Levels of mTOR are increased and ATG7 levels are reduced in DLB brain. Rapamycin, an mTOR inhibitor and autophagy enhancer, is protective in mouse models of neurodegenerative diseases like Alzheimer's disease and PD. Rapamycin reduces a-synuclein accumulation and neurodegenerative phenotype in neuronal cells. Feeding rapamycin diet extends mouse lifespan and the mechanisms are hypothesized to be mediated via delaying age-related diseases including neurodegenerative diseases. The aim of the study is to determine whether long-term feeding rapamycin diet at the dose that extends mouse lifespan attenuates motor deficits in neuronal A53T α-synuclein transgenic mice, which express human A53T α-synuclein richly in brain and spinal cord and develop motor deficits. Mouse diet containing microencapsulated rapamycin (14 ppm in diet; 2.25 mg/kg body weight/day) or the microencapsulation material was fed to age-matched wild type and A53T mice from 13 weeks of age. After 24 weeks of treatment, rapamycin improved performance on forepaw stepping adjustment test, accelerating rotarod test and pole test in both genders of A53T mice. Rapamycin also increased front stride length in male A53T mice. In conclusion, rapamycin attenuated motor deficits in the A53T mice. Further experiments will determine whether the effects of rapamycin are through reducing human α-synuclein expression in brain regions that control and regulate motor function including motor cortex, spinal cord, midbrain, striatum and cerebellum. In addition, it is reported that rapamycin improves myelination in explant cultures from neuropathic mice. Thus, effect of rapamycin on demyelination in A53T mice will also be determined in the brain regions mentioned above.

# Sub article: Impaired locomotor function in ALDH1A1 and GPX1 deficient mice

**Published:** 2012-12-31

**Authors:** Paul Anthony Martinez, Xiang Bai, Margaret Chia-Ying Wey, Vanessa Martinez, Randy Strong, Elizabeth Fernandez

**Affiliations:** 1 Geriatric Research, Education, and Clinical Center, South Texas Veterans Health Care Network, San Antonio, Texas, USA; 2 Department of Pharmacology; 3 Barshop Institute for Longevity and Aging Studies, University of Texas Health Science Center, San Antonio, Texas, USA; 4 Harvard Medical School and Massachusetts General Hospital, Boston, MA, USA

## Abstract

Parkinson's disease (PD) is the second most prevalent neurodegenerative disease. Degeneration of dopamine neurons within the substantia nigra, leads to a substantial decrease in dopamine release in the substantia nigra and the striatum, as well as impaired motor function. Motor symptoms associated with PD include resting tremor, rigidity, and bradykinesia. Although the cause of this disorder remains unclear, several lines of evidence implicate mitochondrial dysfunction and oxidative stress. Major cytosolic enzymes ALDH1 (aldehyde dehydrogenase 1) and GPX1 (glutathione peroxidase 1), are involved in the metabolism of biogenic aldehydes and the reduction of hydrogen peroxide, respectively. ALDH1 is selectively expressed in the midbrain and found to be co-localized with tyrosine hydroxylase within the substantia nigra and ventral tegmental area. Gene profiling studies have been reported showing a decreased expression of ALDH1 in surviving dopaminergic neurons of PD patients. GPX1 gene expression in the substantia nigra of PD patients is also markedly reduced. Therefore, we hypothesize that deletion of both Aldh1a1 and Gpx1 will lead to the accumulation of reactive oxygen species and highly reactive biogenic aldehydes leading to motor deficits. To test this hypothesis, our lab crossed two mouse lines deficient in Aldh1a1 and Gpx1 genes. Here we report impaired locomotor function in Aldh1a1 x Gpx1 knockout mice. These data are consistent with the idea that elevated levels of reactive oxygen species and/or biogenic aldehydes may lead to motor deficits similar to those found in Parkinson's disease.

# Sub article: Link between traumatic brain injury and peripheral nervous system pathology

**Published:** 2012-12-31

**Authors:** Teresa M. Evans, Carlos A. Jaramillo, Holly Van Remmen

**Affiliations:** 1 The Barshop Institute of Longevity and Aging Research; 2 Department of Pharmacology; 3 Department of Cellular and Structural Biology, The University of Texas Health Science Center at San Antonio, TX; 4 Geriatric Research, Education and Clinical Center, South Texas Veterans Health Care System, San Antonio, TX, USA

## Abstract

Traumatic Brain Injury (TBI) is a known risk factor for ALS. The goal of this study is to elucidate the mechanism linking TBI to motor neuron disease, by testing the hypothesis that TBI will accelerate disease progression in animal models of ALS. We used the well-characterized mouse models of familial ALS (G93A SOD1) and sporadic ALS (TDP43, TDP25) to study the effect of TBI on ALS progression. Mice were subjected to a closed head traumatic brain injury and magnetic resonance imaging (MRI) was used 3 days after injury to characterize structural central nervous system pathology and the severity of brain injury. Histological techniques showed neuronal loss (NeuN), astrocyte infiltration (GFAP) and edema (Nissl) following mild TBI in wildtype (WT) and transgenic mice (TG). Our preliminary results indicate that TBI leads to a reduction in grip strength, decreased rotarod performance and muscle denervation via electromyography abnormalities. Also, we have characterized an acceleration of disease related weight loss and overall disease score following TBI in G93A mice. Our results are the first to show that TBI, in an animal model of ALS, results in significantly increased muscle denervation and potentiates disease onset and progression. This work is supported by an individual fellowship grant, 1F31NS080508-01, as well as the Barshop Institute for Longevity and Aging.

# Sub article: Effect of food restriction on sciatic nerve injury associated musculoskeletal loss

**Published:** 2012-12-31

**Authors:** Md M. Rahman, Carmen Rios, Yun Shi, Arunabh Bhattacharya, Marian R. Sabia, Amanda L. Jernigan, Rasel Mohiuddin, Holly Van Remmen

**Affiliations:** 1 Department of Medicine, University of Texas Health Science Center at San Antonio, San Antonio, TX, USA; 2 Barshop Institute for Longevity and Aging Studies, University of Texas Health Science Center at San Antonio, San Antonio, TX, USA; 3 Department of Cellular and Structural Biology, University of Texas Health Science Center at San Antonio, San Antonio, TX, USA; 4 Geriatric Research, Education, and Clinical Center, South Texas Veterans Health Care System, San Antonio, TX, USA

## Abstract

Mechanical inactivity or disuse causes muscle loss and bone loss in both men and women; however, it is not known whether food restriction (FR) has any effect on mechanical inactivity-associated muscle and bone loss. Disuse-associated musculoskeletal atrophy could be associated with nerve injury. The present study aimed to investigate the effect of 40% FR on sciatic nerve injury associated muscle and bone loss and also to analyze if there is any time dependent effect of FR after sciatic nerve injury. Two-month-old male C57BL/6 mice were randomly allocated into two groups: (1) ad libitum (AL) (2) 40% FR fed lab chow for 8 months. The left hind limb of each mouse was then subjected to sciatic nerve crush to induce mechanical inactivity of the particular leg. After different time points (2 days, 7 days, 14 days, 21 days, 28 days and 42 days) of mechanical inactivity, mice were sacrificed and analyzed for muscle mass (wet weight) and bone mass (dual energy x-ray absorptiometry (DXA)). AL fed mice showed significant loss of gastrocnemius and tibia due to mechanical inactivity whereas, FR mice showed protection of both gastrocnemius and tibia from inactivity associated loss. Interestingly, this gastrocnemius and tibial loss protection was stable up to 42 days of mechanical inactivity, we have tested. This data suggests that FR may be beneficial in case of disuse situation commonly happened during aging. Further studies are necessary to determine the musculoskeletal quality and the molecular mechanisms involved in FR mediated protection of musculoskeletal loss due to disuse.

# Sub article: Oxidative stress-dependent impairment of nerve conduction velocity and myelination, potential mechanism of muscle atrophy

**Published:** 2012-12-31

**Authors:** Ryan T. Hamilton, Mike E. Walsh, Yun Shi, Arunabh Bhattacharya, Asish Chaudhuri, Holly Van Remmen

**Affiliations:** 1 Barshop Institute; 2 Department of Cellular and Structural Biology, University of Texas Health Science Center at San Antonio, San Antonio, TX, USA

## Abstract

Oxidative stress is implicated in loss of muscle mass with age in the CuZnSOD deficient mice (Sod1^-/-^). However, the mechanisms of oxidative stress-dependent loss in muscle mass are currently unknown. Since oxidative stress is considered to be an important contributor to muscle atrophy and muscle activity is dependent upon nerve stimulation, this study proposes that oxidative stress damages protein integrity which leads to impaired nerve conduction velocity and myelination. To test our hypothesis, we chose the Sod1^-/-^ mouse model and control c57bl/6 mouse to determine declines in nerve conduction velocity and myelination. Gastrocnemius muscle isolated from the Sod1^-/-^ mice have significant atrophy at 6 and 18 months of age. Sciatic nerve conduction velocity was significantly impaired at both 6 and 18 months of age in the Sod1^-/-^ mice. 6 month old Sod1^-/-^ mice had reduced axon and fiber diameter with what appeared to be changes in myelin morphology which by 18 months of age resulted in reduced myelin thickness and increased g-ratio (axon/fiber diameter). Also, the sciatic nerves from the Sod1^-/-^ mice exhibited significant global increase in protein carbonyls and alteration in exposure of surface hydrophobic domain in proteins. Taken together, these data suggest that loss in nerve conduction velocity and myelin might play a significant functional outcome in gastrocnemius atrophy.

# Sub article: Dietary cholesterol, independent of other types of dietary fat, negatively impacts bone

**Published:** 2012-12-31

**Authors:** Kathryn L. Shultz, Dana A. Godfrey, Cheryl L. Ackert-Bicknell

**Affiliations:** The Jackson Laboratory, Bar Harbor, ME, USA

## Abstract

Half of all Americans over the age of 50 either already have or will develop osteoporosis and osteoporotic fracture is associated with increased mortality rates. Fracture can be considered a chronic condition as complications from fracture can extend well past healing of the initial fracture, thus preventing fracture is required for prolonging healthspan. Bone mineral density (BMD) is highly correlated to fracture risk and environmental factors, such as diet impact BMD. As diet can be modulated, identification of what types of and how dietary constituents decrease BMD will increase our general knowledge about the etiology of osteoporosis and could illuminate opportunities to intercede to prevent fracture. Preliminary studies have suggested that a high fat diet negatively impacts bone mass, but it remains unknown which type of fat mediates these negative effects. In this study, we specifically examined the consequence of increased cholesterol intake on bone mass and osteoblast maturation. We determined that dietary cholesterol, independent of other types of dietary fat negatively impacts on BMD in C57BL/6J female mice. We then established that dietary cholesterol appears to decrease the marrow osteoblast progenitor pools in the femur. In the vertebrae, a high cholesterol diet was associated with a decrease in trabecular bone thickness and with an increase in osteoclastic activity in the vertebrae. Together, this shows that dietary cholesterol, independent of other types of dietary fat, negatively impacts bone mass. In the femur, cholesterol affects the osteoblast linage where as in the vertebrae its effects are mediated via osteoclastic bone resorption.

# Sub article: The effect of age on skeletal muscle stem cells

**Published:** 2012-12-31

**Authors:** Jessica Curtis, Cuong Nguyen, Robert Wersto, Young Jang, Amy Wagers, Julie Mattison, Luigi Ferrucci, Rafael de Cabo

**Affiliations:** 1 Translational Gerontology Branch, National Institute on Aging, Baltimore, MD, USA; 2 Research Resources Branch, National Institute on Aging, Baltimore, MD, USA; 3 Stem Cell Institute, Harvard Medical School, Boston, MA, USA; 4 Longitudinal Study Section, National Institute on Aging, Baltimore, MD, USA

## Abstract

Maintenance of skeletal muscle regenerative capacity is crucial for preservation of muscle mass and function with age. Skeletal muscle precursor (SMP) cells are myogenic stem cells that play a predominant role in muscle regeneration. These cells are located beneath the basal lamina of the myofiber and respond to tissue injury with activation, differentiation and fusion into an existing myofiber. Previous studies have identified a panel of cell surface markers to isolate pure populations of SMP cells from mice with minimal contamination by flow cytometry. Using this technique, the current study assessed the impact of age on SMP content and function. By analyzing male C57Bl/6 mice aged 18–100 weeks on a standard ad libitum diet, it was found that the SMP population decreases by 20–60% with age, depending on the muscle depot. The greatest decline was found in the triceps bracii, which is composed predominantly of Type IIb fibers (fast glycolytic). Furthermore, the regenerative capacity of isolated cells was impaired in older mice, as measured by proliferation and differentiation of SMP cells into myofibers. This study highlights the negative effect of aging on skeletal muscle stem cells. Future work will explore interventions to prevent the loss of regenerative capacity with age.

# Sub article: Determining the role of caspase-2 in osteoclast apoptosis and differentiation

**Published:** 2012-12-31

**Authors:** Danielle A. Victor, Ramaswamy Sharma, Difernando Vanegas, Meenakshi Tiwari, Brian A. Herman

**Affiliations:** UT Health Science Center at San Antonio, Department of Cellular and Structural Biology, San Antonio, TX, USA

## Abstract

Osteoporosis is a silent disease characterized by excess bone resorption by osteoclasts compared to bone formation by osteoblasts. Our lab has shown that old male mice deficient in caspase-2 (Casp2^-/-^), a cysteine protease involved in apoptosis, exhibit severe age-related osteoporosis. Interestingly, these mice also have higher numbers of osteoclasts compared to age-matched wild-type (WT) mice. This could mean that more osteoclasts are being created or less osteoclasts are dying in Casp2^-/-^ animals compared to WT. However, the role of caspase-2 in osteoclasts remains to be elucidated. We hypothesize that caspase-2 plays a dual role in both osteoclast apoptosis and differentiation. With regards to apoptosis, caspase-2 as an upstream component of the apoptotic pathway has been well described in a variety of cell types. Furthermore, cells lacking caspase-2 have been shown to be more resistant to oxidative stressors. Here, we show that osteoclasts derived from Casp2^-/-^ mice are more resistant to 6 hour treatment with various doses of the general stressor H_2_O_2_ and the mitochondrial stressor rotenone compared to osteoclasts from WT mice. Osteoclasts are formed through macrophage fusion that is spurred by the osteoblast-derived cytokines CSF-1 and RANKl. We show that macrophages from Casp2^-/-^ animals form increased numbers of osteoclasts compared to WT. In addition, we have seen that caspase-2 levels decrease in macrophages after RANKl stimulation, suggesting that low caspase-2 expression may be important during osteoclast differentiation. Delineating the role of caspase-2 in the osteoclast may provide new information that will aid in the development of novel osteoporosis treatments.

# Sub article: Synergistic effect of muscle and motor neuron Sod1 on the maintenance of neuromuscular junction structure and function

**Published:** 2012-12-31

**Authors:** Yun Shi, Michael E. Walsh, Daniel A. Pulliam, Ryan T. Hamilton, Yuhong Liu, Yiqiang Zhang, Carlos A. Jaramillo, Holly Van Remmen

**Affiliations:** 1 Barshop Institute for Longevity and Aging Studies; 2 Department of Cellular and Structural Biology; 3 Department of Physiology; 4 Department of Rehabilitation Medicine, University of Texas Health Science Center at San Antonio; 5 GRECC - South Texas Veterans Health Care System, San Antonio, TX, USA

## Abstract

Neuromuscular junction (NMJ) degeneration and muscle atrophy occur with age and in various neuromuscular diseases. Previously we have demonstrated that mice deficient in Cu/Zn superoxide dismutase (CuZnSOD or SOD1) exhibit age-dependent NMJ degeneration, muscle weakness and functional motor deficits. The purpose of this study was 1) to determine whether these changes are associated with alterations in NMJ neurotransmission; 2) to determine whether the NMJ phenotype is a cell-autonomous trait of CuZnSOD deficiency in muscles or neurons. Electrophysiological studies of CuZnSOD knockout mice (KO) demonstrate pathological decrement in compound muscle action potential (CMAP) amplitude with repetitive nerve stimulation (RNS), which is indicative of faulty neurotransmission. To test the second aim, we utilized tissue specific knockout and transgenic mice of SOD1. Neuron-specific SOD1 knockout mice (NKO) developed a moderate reduction in muscle mass, while muscle-specific SOD1 knockout mice (MKO) showed no muscle atrophy. Neither NKO nor MKO mice showed alterations in RNS, suggesting the NMJ deficits in KO mice may be a synergistic effect from both cell types. However MKO mice exhibit multiple characteristics of myopathy including denervation potentials, central nuclei and increased muscle damage upon exercise. It suggests that CuZnSOD plays an essential role in maintaining skeletal muscle integrity. Meanwhile, neuronal SOD1 overexpression rescued muscle atrophy and aberrant CMAP parameters in the KO mice. In conclusion, the complete NMJ phenotype in KO mice is likely caused by deficiency of CuZnSOD in both muscle and neurons. Our data indicate that muscle atrophy in KO mice may be secondary to the neuronal defect.

# Sub article: Is cellular senescence responsible for age-dependent macrophage dysfunction?

**Published:** 2012-12-31

**Authors:** Shoulei Jiang, Carlos J. Orihuela

**Affiliations:** Department of Microbiology and Immunology, University of Texas Health Science Center at San Antonio, San Antonio, TX, USA

## Abstract

Aging is associated with chronic low-grade inflammation, due in part to the pro-inflammatory secretory profile of replicative senescent cells (i.e. senescence associated secretory phenotype [SASP]). Paradoxically, macrophages from aged animals fail to produce the pro-inflammatory cytokines necessary to recruit and activate other immune cells and have poor bacteria killing ability (i.e. age-dependent macrophage dysfunction [ADMD]). A recent publication examining LPS-induced macrophage anergy [Park SH, et al., Nat. Immunol. 2011, 22:12(7): 607–15] triggered us to test the hypothesis that pro-inflammatory cytokines produced by senescent cells may be responsible for ADMD. Bone-marrow derived and J774A.1 macrophages exposed to senescent type II epithelial lung cells (A549 cell line) overnight demonstrated a decreased ability to kill Streptococcus pneumoniae, a gram-positive bacteria and the leading cause of community-acquired pneumonia, versus those exposed to normal cells in vitro. J774A.1 macrophages, but not bone-marrow derived macrophages, exposed to filtered conditioned media from senescent cells also showed an attenuated ability to kill bacteria versus controls. Likewise, they demonstrated an inability to produce de novo Interleukin-6 following stimulation with ethanol-killed pneumococci. Ongoing studies are focused on determining the component produced by senescent cells that is responsible for macrophage dysfunction.

# Sub article: Is proteasome maintenance in the spleen a determinant of sustained health span in the naked mole-rat?

**Published:** 2012-12-31

**Authors:** Karl A. Rodriguez, Rochelle Buffenstein

**Affiliations:** The Sam and Ann Barshop Institute for Longevity and Aging Research and Department of Physiology, University of Texas Health Science Center at San Antonio, San Antonio, TX, USA

## Abstract

The ubiquitin proteasome system (UPS) is responsible for the controlled cleavage of damaged and misfolded proteins and antigen-producing peptides. Commonly reported declines in efficiency of the UPS with age may play a critical role in age-associated dysfunction of protein homeostasis and immune function. The longest-lived rodents, naked mole-rats (Heterocephalus glaber), maintain robust, cancer-free good health for 75% of their 32 year lifespan suggesting that decline in protein homeostasis, observed in other animals, is attenuated or delayed. We compared age-related changes in proteasome activity in whole cell and sub-fractionated lysates from spleen tissues of naked mole-rats and physiologically age-matched mice. Naked mole-rat lysates, as well as cytosolic and nuclear fractions had significantly higher proteasome chymotrypsin-like (ChTL) and trypsin-like (TL) activity than those of age-matched mouse samples. The age-related decline in naked mole-rat ChTL and TL proteasome activity in spleen lysates was negligible; in contrast mice showed a significant age-related decline. By 70% of maximum lifespan proteasome activity of naked mole-rat was unchanged (p > 0.05) whereas mouse declined by 48% (p < 0.02). Similar age-related species differences were observed in all three fractions. Attenuation of age-related UPS decline in naked mole-rats was further supported by sustained maintenance of the 26S proteasome with age, and higher levels of constitutive and immunoproteasome-related proteasome subunits in the naked mole-rat compared to mice. Given the importance of the spleen in immune function, high and sustained UPS in splenic tissue may contribute significantly to prolonged good health in this extraordinary long-lived rodent.

# Sub article: Baboon aging: A model for healthy immune aging?

**Published:** 2012-12-31

**Authors:** Caroline Bonnel, Lourdes T. Arteaga-Cortes, M. Michelle Leland, Peter H. Dube, Ellen Kraig

**Affiliations:** Department of Cellular and Structural Biology, University of Texas Health Science Center, San Antonio, TX

## Abstract

Natural aging processes cause gradual degradation or senescence of the immune system at the humoral and cellular levels. A diminished immune capacity due to aging correlates clinically with decreased vaccine efficacy and increased susceptibility to infection and cancer. Due to this loss in immunity the protective capacity of new vaccines should be determined in older individuals. Animal models for vaccine development should embody the immunosenescent effects observed in aging humans. A baboon model was tested by immunizing young (5–6 years of age) and old (17–22 years) animals with the LcrV and F1 candidate vaccine antigens from Yersinia pestis. Contrary to the expected loss of immunity, older baboons demonstrated high antibody titers and exhibited strong T cell proliferation, particularly in response to LcrV. These findings suggest that aging has less effect on the baboon immune system. The cellular and cytokine responses to antigen stimulation were measured to better characterize the effects of aging on T cell fine specificity. T cell proliferation and IFN-γ ELISpots were used to map which of 32 overlapping synthetic F1 peptides stimulated T cells from the immunized baboons. Spectratype analysis of T cell receptor (TCR) expression indicates no age associated loss in T cell activation of the overall repertoire diversity. Currently, F1-specific T cell lines are being generated using herpesvirus papio transformed B cell lines as antigen presenting cells. Future efforts will focus on characterizing the TCR repertoire of an F1-speific response.

# Sub article: Calorie restriction retards growth of ethylnitrosourea-induced glioma in rats

**Published:** 2012-12-31

**Authors:** Maddie Roman, L.C. Flores, M. Ortiz, S. Dube, Y. Zhang, S. Lee, G.B. Hubbard, Y. Ikeno

**Affiliations:** 1 Barshop Institute for Longevity and Aging Studies; 2 Department of Physiology; 3 Department of Pathology, University of Texas Health Science Center at San Antonio; 4 Research Service, GRECC, Audie Murphy VA Hospital, San Antonio, TX, USA

## Abstract

The anti-tumor action of calorie restriction (CR) and the possible underlying mechanisms on tumor growth were investigated using ethylnitrosourea (ENU)-induced glioma in rat. ENU was given transplacentally at gestational day 15. The brain from 4, 6, and 8-month-old rats fed either ad libitum (AL) or calorie restricted diets (40% restriction of total calories compared to AL rats) were studied. Tumor burden was assessed by comparing the size and number of gliomas present in the brain. Immunohistochemical analysis was used to detect the lipid peroxidation products [4-hydroxy-2-nonenal (HNE), malondialdehyde (MDA), and acrolein] and nitrotyrosine to document oxidative stress, levels of glycated end products, cell proliferation activity (PCNA), and cell death (ssDNA) associated with the development of gliomas. The results showed that the number of gliomas did not change with age in the AL groups; however, the average size of the gliomas was significantly larger in the 8-month-old group compared to that of the younger groups. Immunopositive areas for HNE, MDA, acrolein, nitrotyrosine, and glycated end products increased with the growth of gliomas. The CR group showed both reduced number and size of gliomas, less accumulation of oxidative damage, and less glycated end products compared to the AL group. Furthermore, the CR group showed less PCNA positive and more ssDNA positive cells. Interestingly, we also discovered that the anti-tumor effects of CR were associated with less accumulation of hypoxia inducible factor-1α (HIF1α) levels and a reduction in the mammalian target of rapamycin (mTOR) signaling. Our results are very exciting because they could not only demonstrate the anti-tumor effects of CR on oligodendroglioma, but also indicate the possible underlying mechanisms, i.e., anti-tumor effects of CR could be mediated by the changes in redox-sensitive and/or nutrient sensing signaling pathways. (Supported by grants from the VA Merit Review, the American Federation for Aging Research, the Glenn Foundation, and San Antonio Nathan Shock Center)

# Sub article: The effects of thioredoxin 1 down-regulation and/or thioredoxin 2 overexpression on aging and cancer

**Published:** 2012-12-31

**Authors:** Melanie Ortiz, L.C. Flores, S. Dube, M. Roman, A. Bhattacharya, Y. Zhang, A. Salmon, W. Qi, Y. Liu, S. Lee, Holly Van Remmen, A. Richardson, G.B. Hubbard, Y. Ikeno

**Affiliations:** 1 Barshop Institute for Longevity and Aging Studies; 2 Department of Physiology; 3 Department of Pathology; 4 Cellular and Structural Biology, Molecular Medicine; 5 University of Texas Health Science Center at San Antonio; 6 Research Service, GRECC, Audie Murphy VA Hospital, STVHCS, San Antonio, TX, USA

## Abstract

Our laboratory has conducted the first detailed study on the effect of overexpressing or down-regulating thioredoxin 1 (Trx1: cytosol) or thioredoxin 2 (Trx2: mitochondria) on aging. Interestingly, we found that the Trx2Tg mice showed an extension of median lifespan compared to wild-type mice, although we observed little increase in survival of the Trx1Tg mice. The extension of lifespan of Trx2Tg mice was correlated to less reactive oxygen species (ROS) production from mitochondria and less oxidative stress. These data show that overexpressing Trx in the mitochondria may be more important than in the cytosol on aging because mitochondria are a major source of ROS. When we tested the effects of reduced levels of Trx in cytosol or mitochondria on aging, we surprisingly observed the reversed effects, i.e., an increase in survival of the Trx1KO mice compared to wild-type mice, while the Trx2KO mice showed little effects on lifespan. The extension of lifespan of Trx1 KO mice was associated with less cancer compared to wild-type mice at 22–24 months of age. These results indicate that reduced cancer in the Trx1KO mice could be one of the contributing factors of extended lifespan. Our data are exciting in that we show 1) overexpressing Trx in the mitochondria increases lifespan, but overexpressing Trx in the cytosol has little effect on lifespan, which is similar to the results of mCAT mice; and 2) down-regulating Trx in the cytosol increases lifespan and reduces cancer, but down-regulating Trx in mitochondria has no effect on lifespan or cancer. These paradoxical, but intriguing results could indicate that the Trx2Tg and Trx1KO mice attenuate aging through different mechanisms, e.g., protection of mitochondria against oxidative stress and reduced age-related pathology, e.g., cancer. (Supported by grants from the VA Merit Review, the American Federation for Aging Research, and the Glenn Foundation)

# Sub article: Cytoprotection and healthy aging in the naked mole-rat

**Published:** 2012-12-31

**Authors:** Kaitlyn N. Lewis, Rochelle Buffenstein

**Affiliations:** Department of Cellular and Structural Biology, Department of Physiology, and Barshop Institute for Longevity and Aging Studies, STCBM Building, San Antonio, TX, USA

## Abstract

Long-lived animal models across multiple phyla have a marked resistance to toxins and other xenobiotics. The longest-lived rodent, the naked mole-rat, has a maximum lifespan of 32 years and is the size of mouse yet lives almost 8 times longer. During their very long lifespan, naked mole-rats show minimal declines in many physiological and molecular age-related characteristics, and most interestingly, an incidence of spontaneously occurring cancer has never been reported. Naked mole-rats are also very resistant to an extensive array of toxins in vitro. We hypothesize that cytoprotective mechanisms in this species are contributing to their protection. We focus on pathways regulated by nuclear factor-erythroid 2-related factor-2 [Nrf2] as the key cytoprotective signaling pathway facilitating this broad resistance to cytotoxins and stressors. This ubiquitously expressed and highly conserved transcription factor has been heavily researched with regards to toxin resistance and cancer, and has been shown to interact with p21 and tumor suppressor p53, implying a role for Nrf2 in cell cycle regulation and cancer progression. Naked mole-rats show an in vitro and in vivo constitutive upregulation of Nrf2-cytoprotective signaling as well as resistance to toxins in both fibroblasts and whole animals. These long-lived rodents also show pronounced resistance to carcinogenesis in vivo and our data reveal that oncogenic and apoptotic activation may be more sensitive in naked mole-rats. By utilizing the naked mole-rat as a model of impeccable healthspan and lengthened lifespan, we may not only identify novel mechanisms that contribute to toxin resistance and cancer prevention, but also longevity.

# Sub article: Epigenetic modifiers and modifications mediate the effects of dietary restriction

**Published:** 2012-12-31

**Authors:** Michael E. Walsh, Arunabh Bhattachrya, Holly Van Remmen

**Affiliations:** 1 Department of Cellular and Structural Biology; 2 Barshop Institute for Longevity and Aging Studies, The University of Texas Health Science Center at San Antonio; 3 South Texas Veterans Health Care System, San Antonio, TX, USA

## Abstract

The molecular mechanisms behind aging are complex, and one emerging theory asserts that aging occurs as a result of changes in the epigenetic landscape. Here we test the hypothesis that dietary restriction (DR) mediates its anti-aging effects through epigenetic modifiers and modifications. To test the hypothesis that DR mediates its protective effects through epigenetic modifiers, we used surgical nerve crush to model the denervation that occurs in aging skeletal muscle. We demonstrate that DR, even when initiated after surgery, protects against denervation-induced muscle atrophy as measured by gastrocnemius wet weight. DR inhibited the induction of histone deacetylase 4 (HDAC4), a known mediator of atrophic signaling in skeletal muscle. Using the general HDAC inhibitor sodium butyrate (NaBu), we demonstrate that pharmacologically inhibiting HDACs protects against the muscle loss induced by nerve crush, thus mimicking the effects of dietary restriction. To investigate the effects of aging and DR on histone modifications, we analyzed liver histones from young and old animals fed ad libitum or dietary restricted for acetylation at specific residues. We found an age-related decrease in histone H3K9 acetylation, and importantly this decrease was prevented by dietary restriction. To simulate the increase in histone acetylation seen with dietary restriction, we fed old animals the HDAC inhibitor NaBu which resulted in reduced fat mass and increased glucose tolerance over time, consistent with known effects of dietary restriction. Overall, our data support the epigenetics theory of aging and indicates that dietary restriction uses epigenetic mechanisms to protect against age-related pathologies. (This work was funded by the UTHSC at San Antonio Biology of Aging Training Grant to Steve N. Austad (MEW T32AG021890-10).

# Sub article: Protein homeostasis in the longest lived animal

**Published:** 2012-12-31

**Authors:** Stephen Treaster, Keith Maslin, Iain Ridgway, Asish Chaudhuri, Steven Austad

**Affiliations:** 1 Barshop Institute for Longevity and Aging Studies; 2 Department of Cellular and Structural Biology; 3 Department of Biochemistry; 4 Department of Molecular Medicine, The University of Texas Health Science Center at San Antonio, San Antonio, TX, USA; 5 School of Ocean Sciences, Bangor University, Wales, UK

## Abstract

Protein homeostasis has been implicated in the aging process in a variety of model organisms. We are utilizing a range of marine bivalve mollusk species, with lifespans ranging from under a decade to over five hundred years, in a comparative study to investigate the hypothesis that long life requires superior proteome stability. These ages can be individually determined by counting growth rings in the shell. This experimental system provides a unique opportunity to study closely related organisms with vastly disparate longevities, including the longest lived animal, and their relative proteome stabilities. Specifically, we are testing their ability to maintain structure and function under various stressors, as well as prevent protein damage and aggregation. Furthermore, the influence of each species’ isolated metabolite fraction is being investigated on each of these proteostasis aspects. Protein damage and unfolding were quantified by incorporation of two fluorescent probes, specific for carbonyls and exposed hydrophobic surfaces. Preservation of function was measured by representative enzyme activity, such as GAPDH, when stressed in-vitro. Stress induced aggregation of both endogenous proteins and exogenous, aggregation prone bait proteins were also. The bait proteins used include amyloid beta, the aggregation prone peptide associated with Alzheimer's disease. The macromolecules facilitating enhanced proteostasis in the longest lived animal species could have dramatic importance to various age-related protein diseases.

# Sub article: Rapamycin has differential effects than Dietary Restriction in the transcriptome and metabolome of young mice

**Published:** 2012-12-31

**Authors:** Wilson C. Fok, Alex Bokov, Jon Gelfond, Yiqiang Zhang, Mark Doderer, Yidong Chen, Martin Javors, Bill Wood, Yongqing Zhang, Kevin Becker, Arlan Richardson, Viviana Perez

**Affiliations:** 1 Department of Cellular and Structural Biology; 2 Barshop Institute for Longevity and Aging Studies; 3 Department of Epidemiology & Biostatistics; 4 Department of Physiology; 5 Greehey Children's Cancer Research Institute; 6 Cancer Therapy and Research Center; 7 Department of Psychiatry, The University of Texas Health Science Center at San Antonio, San Antonio, TX, USA; 8 National Institute on Aging, Baltimore, MD, USA; 9 Research Service, Audie Murphy VA Hospital (STVHCS), San Antonio, TX, USA; 10 Department of Biochemistry and Biophysics, Linus Pauling Institute, Oregon State University, Corvallis, OR, USA

## Abstract

Rapamycin (Rapa) and dietary restriction (DR) are two manipulations consistently shown to increase the lifespan of mice. To investigate whether Rapa and DR affect similar pathways in mice, we compared the effects of feeding mice ad libitum (AL), Rapa, DR, or a combination of Rapa and DR (Rapa + DR) on the transcriptome and metabolome of the liver. The principle component analysis of the transcriptome shows that Rapa and DR are distinct groups. Of the 2724 genes that significantly change with either Rapa or DR compared to mice fed AL, 79% are unique to DR or Rapa; only 21% of the genes are common to DR and Rapa. A similar observation was made when genes were grouped into pathways by Ingenuity Pathway analysis; 76% of the pathways are uniquely changed by DR or Rapa. The metabolome shows an even greater difference between Rapa and DR; only 6% of the metabolites that change significantly from AL mice are common to Rapa and DR. Interestingly, the number of genes significantly changed in Rapa + DR mice compared to AL mice was twice as large as the number of genes significantly altered by either DR or Rapa. In summary, while both Rapa and DR increase lifespan, their global effect on liver is quite different and a combination of Rapa and DR results in alterations in a large number of genes that are not significantly changed by either manipulation alone.

# Sub article: Tale of new technologies: Protein thiol homeostasis, protein carbonylation and conformation. Application in biomedical research

**Published:** 2012-12-31

**Authors:** Asish Chaudhuri, Rochelle Wei

**Affiliations:** The Sam and Ann Barshop Institute for Longevity and Aging Studies and Biochemistry, University of Texas Health Science Center at San Antonio, San Antonio, TX, USA

## Abstract

A key component of my research is to develop new generations of techniques to understand how oxidative stress-mediated protein oxidation and perturbation of functional structure contribute to aging and age-related diseases. Over the past nine years, I have been actively involved in developing techniques related to measurement of protein oxidation and conformational changes that occur during aging and in disease conditions (Chaudhuri et al. 2001, 2006; Pierce et al. 2006, 2008; Perez et al. 2009; Salmon et al. 2009; Perez et al. 2010; Bhattacharya et al. 2011; Wei et al. 2012). One of the common and unique aspects of all these technologies is the use of fluorescent molecules as probes to detect changes in protein oxidation and conformation. As fluorescent probes in general give high quantum yield, it helps to identify and quantify the potential target proteins that are present in low level and have subtle changes in conformation in any patho-physiological condition. Development of these techniques is an important part of biological research considering the fact that the oxidative stress plays an important role in aging and various diseases including Alzheimer's, Parkinson's, ALS, cancer, heart disease, arthritis, etc. As a result, many investigators are interested in determining the underlying mechanism of the role of imbalanced protein thiol homeostasis; protein oxidation and alteration of conformation contribute to aging and diseases. Most importantly, researchers want to determine if the imbalanced protein homeostasis can be modulated by experimental manipulations, such as calorie restriction and pharmacological intervention. These new sets of techniques will give investigators the necessary tools to delve into the molecular mechanisms involved in aging and age-related diseases.

# Sub article: Role of caspase-2 as an endogenous repressor of autophagy

**Published:** 2012-12-31

**Authors:** Meenakshi Tiwari, Lokendra K. Sharma, Difernando Vanegas, Yidong Bai, Brian Herman

**Affiliations:** Department of Cellular and Structural Biology, University of Texas Health Science Center at San Antonio, STRF, San Antonio, TX, USA

## Abstract

Caspase-2 has been shown to play a role in aging, neurodegeneration and cancer. The contributions of capase-2 have been attributed to its regulatory role in apoptotic and non-apoptotic processes including cell-cycle, DNA repair, lipid biosynthesis, and regulation of oxidant levels in cells. Recently, our lab demonstrated that caspase-2 modulates autophagy during oxidative stress. Here we report the novel finding that caspase-2 is an endogenous repressor of autophagy. Knockout (KO) or knockdown of caspase-2 resulted in upregulation of autophagy in variety of cell types and tissues. Reinsertion of caspase-2 in caspase-2-knockout mouse embryonic fibroblast (MEF's), suppressed autophagy suggesting its role as a negative regulator of autophagy. Loss of caspase-2-mediated autophagy involved down regulation of mTOR and upregulation of AMPK activation; knocking-down of AMPK1/2 inhibited autophagy. Interestingly, siRNA-mediated knockdown of ATG5 and ATG7 failed to inhibit autophagy induced by the loss of caspase-2 suggesting involvement of the non-canonical pathway of autophagy. Our results also indicate involvement of enhanced intracellular reactive oxygen species levels, down regulation of p38 and upregulation of ERK/MEK activation in autophagy-induction due to loss of caspase-2. In response to a variety of apoptotic stimuli that induce caspase-2-mediated apoptosis, caspase-2-KO cells demonstrated further upregulation of autophagy compared to WT MEFs. Enhanced autophagy improved the survival of caspase-2-deficient cells, which maintained high ATP levels. In conclusion, we document a novel role for caspase-2 as a negative regulator of autophagy, which may provide important insight into the role of caspase-2 in aging, neurodegeneration and cancer. The current findings are the first to provide evidence for regulation of caspase activity by autophagy and thus broaden the molecular basis for the observed polarization between autophagy and apoptosis.

# Sub article: Molecular events in kidney matrix metabolism in aging mice

**Published:** 2012-12-31

**Authors:** Kavithalakshmi Sataranatarajan, Denis Feliers, Meenalakshmi M. Mariappan, Hak Joo Lee, Myung Ja Lee, Robert T. Day, Himabindu Yelamanchili, Goutam Ghosh Choudhury, Jeffrey L. Barnes, Holly Van Remmen, Arlan Richardson, Balakuntalam S. Kasinath

**Affiliations:** 1 Department of Medicine; 2 The Barshop Institute for Longevity and Aging Studies, University of Texas Health Science Center, San Antonio, TX, USA; 3 Geriatric Research, Education and Clinical Center; 4 South Texas Veterans Health Care System, San Antonio, TX, USA

## Abstract

C57BL6 mice were studied in youth (4–6 mo), middle-age (18 mo) and old-age (26–32 mo). Albuminuria increased, and, rise in serum cystatin C indicated that renal clearance function fell with aging; there was marked heterogeneity. Kidney hypertrophy and expansion of glomerular and tubulo-interstitial matrix were progressive. Increased mRNA correlated with increase in type III collagen in middle-aged and old mice, suggesting transcriptional regulation. In old-age, increase in mRNA correlated with type I collagen protein; however, in middle age, type I collagen was increased despite unchanged mRNA. Data from ChIP analysis of binding of transcription inhibitors ZEB1/ZEB2 to the type Iα2 promoter, and, polysome assay for mRNA translation did not explain type I collagen increase in middle-age. Thus, decreased degradation could lead to type I collagen increment in middle-age. Matrix changes coincided with TGFβ/SMAD3 activation. SMAD3 binding to collagen type Iα2 promoter was increased. Since microRNAs (miRs) control protein synthesis, we studied TGFβ-regulated miRs. The renal cortical content of miR-21 and miR-200c was increased but that of miR-192, miR-200a or miR-200b was unchanged suggesting selectivity. Increase in miR-21 and miR-200c was associated with reduced expression of their targets, Sprouty-1 and ZEB2, respectively; another miR-21 target, PTEN, was unchanged. Sprouty and ZEB2 inhibit growth factor signaling and expression of miR-21, respectively. Conclusion: Distinct transcriptional and post-transcriptional mechanisms contribute to kidney matrix protein increment in middle and old age. Kidney integrity is essential for maintenance of health span. Understanding mechanisms contributing to renal senescence could identify targets for intervention to improve health span.

# Sub article: The development of a new mouse model to study the role of mTOR in aging and age-associated diseases: the TSC1 transgenic mice

**Published:** 2012-12-31

**Authors:** Yiqiang Zhang, Mike Walsh, Yuji Ikeno, Vivian Diaz, Tyler Curiel, Merry Lindsey, Holly Van Remmen, Arlan Richardson

**Affiliations:** 1 Barshop Institute; 2 Department of Physiology; 3 Cellular and Structural Biology; 4 Pathology and Medicine; 5 Cancer Therapy and Research Center; 6 The Cardiovascular Proteomics Center, The University of Texas Health Science Center at San Antonio, San Antonio, TX, USA; 7 Geriatric Research, Education, and Clinical Center, South Texas Veterans Health Care System, San Antonio, TX, USA

## Abstract

The development of animal models targeting different components of the TOR signaling pathway has accelerated our understanding of the role of mTOR in animal development, metabolism, diseases, and aging. In addition, the discovery of mTOR inhibitors has further enhanced our ability to define the role of mTOR signaling in various patho-physiological conditions and to develop therapeutic strategies for the treatment of different diseases. Here, we report the development and characterization of a new mouse model, which overexpresses TSC1 (named Tsc1^tg^ mice), part of the mTOR inhibitor complex TSC1/2 (tuberous sclerosis complex 1/2). Overexpression of TSC1 stabilized TSC2 and inhibited mTOR signaling in most tissues including the heart, liver, kidney, skeletal muscle and spleen. The levels of several important cell signaling pathways were found altered in Tsc1^tg^ mice. The body weight of Tsc1^tg^ mice exhibits slight gender difference, with significant increase in male mice at both young and advanced ages while only slight increase in female mice at both ages, when compared to age-matched wild type littermates. Body composition of Tsc1^tg^ mice exhibits an age-associated change; with significantly higher fat mass but lower lean mass at advanced ages. At 4–8 months of age, Tsc1^tg^ mice have normal cardiac function as measured by echocardiography. But, when challenged with isoproterenol, Tsc1^tg^ mice developed less cardiac hypertrophy than age-matched wild type littermates. Importantly, Tsc1^tg^ mice performed significantly better with treadmill test. Finally, the immune response of Tsc1^tg^ mice exhibit subtle changes over wild type control mice. In conclusion, this model will be very useful to study the role of mTOR in such diseases that are associated with a deregulation of mTOR signaling, including cancer, cardiovascular diseases, and metabolic disorders. It will also be an interesting model to study the role of mTOR in mammalian aging, complementary to the rapamycin-feeding approach.

# Sub article: Sex differences in response to rapamycin. Implications for senescence retarding therapies

**Published:** 2012-12-31

**Authors:** Vanessa Soto, Alex Bokov, John Gelfond, Lauren Sloane, Vivian Diaz, Keith Maslin, Stephen Treaster, Arlan Richardson, Steven Austad, Kathleen Fischer

**Affiliations:** 1 Barshop Institute; 2 Department of Physiology; 3 Department of Cellular and Structural Biology; 4 Department of Molecular Medicine; 5 Department of Biostatistics and Epidemiology; 6 Department of Pathology, University of Texas Health Sciences Center at San Antonio, TX; 7 Geriatrics Research, Education and Clinical Center, South Texas Veterans Health Care System, San Antonio, TX; 8 State University of New York, Delhi, NY

## Abstract

Sex differences in life and health span are ubiquitous in humans. Women in the developed world live longer than men even if heart disease, the number one cause of death in men, were completely eliminated. Analogously, female mice respond better to a number of senescence-retarding genetic or pharmacological interventions. Particularly notable in this respect, inhibition of TOR signaling via deletion of S6K1 improves both life- and health span in female mice but has no discernible effect in males. Here we show that aging male and female C57BL/6 mice respond to rapamycin in an age and sex-specific manner. There is a larger and more robust effect on longevity in females compared with males and measures of health span have multiple age and sex-specific effects. Age, sex and age · sex-specific differences in body composition, rotarod performance, gait, measures of activity, sleep and metabolism were observed in animals treated with enteric rapamycin (=e-rapa) relative to controls. There has been very little research on the basic biological mechanisms involved in sex differences in aging, in part because past research suggested that laboratory mice and rats do not show clear consistent trends in sex-specific longevity or health span. Our results suggest that sex differences in some measures of mouse health span may only become apparent late in life and that there are sex-specific responses to senescence-retarding treatments that merit further exploration.

# Sub article: Stress resistance in the long-lived Surf1 knockout mouse

**Published:** 2012-12-31

**Authors:** Daniel Pulliam, Shuana Hill, Wenbo Qi, Adam Salmon, Deepa Sathyaseelan, Holly Van Remmen

**Affiliations:** 1 Barshop Institute for Longevity and Aging Studies; 2 Department of Cellular and Structural Biology, University of Texas Health Science Center San Antonio; 3 GRECC South Texas Veteran Health Care System, San Antonio, TX, USA

## Abstract

Loss of mitochondrial function with age has been implicated as an influencing factor in the aging process. However, studies from model organisms ranging from yeast to mammals have shown that moderate disruption of the electron transport chain can enhance longevity. In the Surf1 knockout mouse, there is a 50–75% decline in cytochrome c oxidase (complex IV of the electron transport chain) activity and a 20% extended median lifespan. Previous studies of fibroblasts from long-lived rodents have shown a correlation between increased resistance to cellular stresses and longevity. Here we investigate whether fibroblasts from Surf1 knockout animals are more resistant to stress than wild type controls. Interestingly, these results are dependent upon the passage of the cells. Early passage (<2) cells show little difference between Surf1 KO and control cells. However, in later passaged fibroblasts, KO cells are significantly more resistant to paraquat, a superoxide radical generator, than control fibroblasts. Additionally, in vivo administration of diquat (50mg/kg), a superoxide radical generator, resulted in a significantly attenuated increase in markers of oxidative stress. Animals sacrificed six hours post diquat treatment resulted in a 24% increase in liver F2-isoprostanes in wild type animals. However, in the Surf1 KO livers the increase in F2-isoprotanes was 15%. This suggests that Surf1 KO mice are more resistant to exogenous oxidative stress than wild type control animals. (This work is supported by a grant from the Ellison Medical Foundation.)

# Sub article: Metformin improves health span and lifespan in mice

**Published:** 2012-12-31

**Authors:** Alejandro Martin-Montalvo, Evi M. Mercken, Sarah J. Mitchell, Hector H. Palacios, Michel Bernier, Rafael de Cabo

**Affiliations:** Laboratory of Experimental Gerontology, National Institute on Aging, National Institutes of Health, Baltimore, MD, USA

## Abstract

Metformin, a drug commonly prescribed to treat type-2 diabetes, has been found to extend healthspan, delay cancer incidence and progression and to increase lifespan in laboratory animals. We show here that treatment with metformin (0.1% w/w in diet) starting at one year of age extends healthspan and lifespan in male mice, while a higher dose (1% w/w) was toxic. Treatment with metformin mimicked some of the benefits of calorie restriction, such as improved physical performance, increased insulin sensitivity, and reduced LDL and cholesterol levels without a decrease in caloric intake. At a molecular level, metformin increased AMP-activated protein kinase activity and increased antioxidant protection, resulting in reductions in both oxidative damage accumulation and chronic inflammation. Our results indicate that these actions may contribute to the beneficial effects of metformin administration on health span and lifespan. These findings are in agreement with current epidemiological data and raise the possibility of metformin-based interventions to promote healthy aging.

# Sub article: Epigenetic regulation of mid-and late-life longevity in drosophila

**Published:** 2012-12-31

**Authors:** Philip McDonald, Brian M. Maizi, Robert Arking

**Affiliations:** Department of Biological Sciences, Wayne State University, Detroit, MI, USA

## Abstract

We tested the effects of two Class I histone deacetylase inhibitors (HDAcI) on the longevity of normal-lived (Ra) and long-lived (La) strains of Drosophila melanogaster. Only deleterious effects are noted when the first HDAcI tested (sodium butyrate, NaBu) is fed to the La strain at any developmental or adult stage. When fed to the Ra strain, this drug also has negative effects when administered over the entire larval and/or adult life span, or when administered over the adult health span only. Importantly, however, it significantly decreases mortality rates and increases longevity when administered only in the adult transition or senescent spans. A different HDAcI (suberoylanilide hydroxamic acid, SAHA) administered to the same strain also showed significant late-life extending effects, suggesting that this is not an isolated effect of one drug. These results suggest that the stage-specific gene regulatory mechanisms affected by NaBu or SAHA are those intimately involved in inducing gene expression patterns characteristic of a healthy senescence. Epigenetically active molecules, if given at the appropriate stage, allow the fly to shift from a senescent span characterized by a high age-specific mortality rate to one with a lower age-specific mortality rate. These studies may provide an experimental basis with which to shed light on the fraility syndrome affecting some aging organisms.

# Sub article: Curcumin is an early-acting stage-specific inducer of extended functional longevity in drosophila

**Published:** 2012-12-31

**Authors:** Soh Jung-Won, Nicholas Marowsky, Thomas J. Nichols, Abid M. Rahman, Tayaba Miah, Paraminder Sarao, Rawia Khasawneh, Archana Unnikrishnan, Ahmad R. Heydari, Robert B. Silver, Robert Arking

**Affiliations:** 1 Department of Biological Sciences; 2 Nutrition & Food Sciences; 3 Pharmacology, Wayne State University, Detroit, MI, USA

## Abstract

Feeding larvae of a normal-lived strain, but not a long-lived strain, with curcumin induces an extended adult health span with significantly increased median and maximum longevities. This phenotype shows no additive effect on longevity when combined with an adult dietary restriction (DR) diet, suggesting that curcumin and DR operate via the same or overlapping pathways for this trait. This treatment significantly slows the age-specific mortality rate so that it is comparable with that of genetically selected long lived animals. The larval treatment also enhances the adults’ geotactic activity in an additive manner with DR, suggesting that curcumin and DR may use different pathways for different traits. Feeding the drug to adults during only the health span also results in a significantly extended health span with increased median and maximum life span. This extended longevity phenotype is induced only during these stage-specific periods. Feeding the drug to adults over their whole life results in a weakly negative effect on median longevity with no increase in maximum life span. There are no negative effects on reproduction, although larval curcumin feeding increases development time; but it apparently accelerates the normal late-life neuromuscular degeneration seen in the legs. Gene expression data from curcumin-fed larvae shows that the TOR pathway is inhibited in the larvae and the young to midlife adults, although several other genes involved in longevity extension are also affected. These data support the hypothesis that curcumin acts as a stage-specific DR mimetic neutriceutical; and suggest that the search for DR mimetics may be enhanced by the use of stage-specific screening of candidate molecules.

# Sub article: Examination of possible signaling pathways which lead to longevity modulation in C. elegans

**Published:** 2012-12-31

**Authors:** Robert J. Mishur, Joshua C. Judkins, Jeffrey A. Butler, Parag Mahanti, Frank C. Schroeder, Shane L. Rea

**Affiliations:** 1 University of Texas Health Science Center at San Antonio, The Barshop Institute for Longevity and Aging Studies, San Antonio, TX, USA; 2 Cornell University, Boyce Thompson Institute, Ithaca, NY, USA

## Abstract

Mitochondrial mutations in Caenorhabditis elegans can lead to either a shortening of lifespan or, unexpectedly, lifespan extension. Long-lived mitochondrial mutants (Mit mutants) live twice as long as wild-type animals, have delayed development, and reduced adult sizes. We have used a GC-MS-based metabolic footprinting approach to show that Mit mutants employ a common metabolism, distinct from wild-type animals and from short-lived mitochondrial mutants. The hallmark feature of the Mit metabolism is overproduction of pyruvate and various branched-chain ketoacids. We postulate that these compounds may act as mitokines, signaling molecules emanating from the mitochondria, to result in organismal lifespan extension. We have shown that at least four compounds found in the exometabolome of the Mit mutants can delay development when administered to wild type animals. At least one of these compounds, pyruvate, has also previously been reported to increase lifespan when fed to worms. Lifespan studies on the remaining compounds are underway. We have recently begun additional studies to determine whether the Mit mutants also produce a characteristic profile of ascarosides. Ascarosides are small signaling molecules based on the dideoxysugar ascarylose, and compose the pheromone which signals dauer development in C. elegans. This alternate larval state is resistant to stress and is considered non-aging, since upon leaving the dauer state animals live out their normal lifespan. Interestingly, we found a complete absence of one ascaroside in short-lived mitochondrial mutants. Experiments are underway to determine whether this molecule is capable of recovering lifespan in these mutants.

# Sub article: Are DBA/2 mice truly resistant to calorie restriction?

**Published:** 2012-12-31

**Authors:** Sarah J. Mitchell, Theresa M. Ward, Hector Palacios, Robin K. Minor, Rafael de Cabo

**Affiliations:** 1 Laboratory of Experimental Gerontology, National Institute on Aging, National Institutes of Health, Baltimore, MD, USA; 2 Kolling Institute of Medical Research, Royal North Shore Hospital and the University of Sydney, NSW, Australia; 3 Sydney Medical School, The University of Sydney, Sydney, NSW, Australia

## Abstract

Emerging evidence suggests that both diet composition and genetic make-up have a key role in the beneficial effects of calorie restriction (CR). CR-mediated improvements in health and/or longevity may not be universal, even within species. Furthermore the responsiveness to CR may depend on subtleties of the treatment protocol, diet composition or the “intensity of CR”. In this study we determined the differential response to CR levels of DBA/2J mice. We are testing two main hypotheses: (i) that a milder CR intervention will provide beneficial effects on lifespan and healthspan in DBA/2 mice and (ii) regardless of the lack of effect on longevity, there are healthspan benefits even at the higher CR level. Male and female DBA/2J and C57BL/6 mice on one of three diets: ad libitum (AL), 20% CR, 40% CR starting at 6 months of age. Preliminary data indicates that in female mice there is no difference in median lifespan extension between 20% and 40% CR. In male mice it appears that 20% CR is more beneficial in extending median lifespan. Insulin levels are significantly lower in all DBA mice compared to their C57BL/6 counterparts. CR lowers insulin levels in all groups. We observed a stepwise decrease in insulin resistance with increasing CR, but only in males. In female mice, there was no difference in insulin levels between 20% or 40% CR groups. These results indicate that DBA/2J do respond to CR and supports the idea that there is an “ideal” CR dose for a particular strain.

# Sub article: Preliminary results from the health span study: First steps toward a rigorous definition of healthy aging in C57BL/6 mice

**Published:** 2012-12-31

**Authors:** Alex F. Bokov, Jon A. Gelfond, Lauren B. Sloane, Kathleen E. Fischer, Keith P. Maslin, Samantha Rendon, Vanessa Soto, Salvatore Oddo, Smita Majumder, Balakuntalam S. Kasinath, Kavitha L. Satara Natarajan, Babatunde O. Oyajobi, Anjana Gupta, Brandon W. McCluskey, Holly Van Remmen, Michael E. Walsh, Merry L. Lindsey, Arlan G. Richardson, Steven N. Austad

**Affiliations:** 1 Department of Epidemiology & Biostatistics, The University of Texas Health Science Center at San Antonio, San Antonio, TX, USA; 2 Department of Biology, State University of New York at Delhi, Delhi, NY, USA; 3 Barshop Institute for Longevity and Aging Studies, The University of Texas Health Science Center at San Antonio, San Antonio, TX, USA; 4 Department of Physiology, The University of Texas Health Science Center at San Antonio, San Antonio, TX, USA; 5 Department of Cellular and Structural Biology, The University of Texas Health Science Center at San Antonio, San Antonio, TX, USA; 6 Research Service, Audie Murphy VA Hospital, South Texas Veterans Health Care System, San Antonio, TX, USA; 7 Department of Molecular Medicine, The University of Texas Health Science Center at San Antonio, San Antonio, TX, USA; 8 Department of Medicine, The University of Texas Health Science Center at San Antonio, San Antonio, TX, USA; 9 Lab Animal Resources, The University of Texas Health Science Center at San Antonio, San Antonio, TX, USA

## Abstract

The Health Span Study data are an unprecedented cross-sectional window into the biology of rodent aging, and our newly-developed Health Span Database makes it possible to organize, curate, share, and analyze this information in ways that would have otherwise not been practical. Here we present a range of measurements (e.g. body composition, grip strength, and gait analysis) that significantly change with chronological age of the animal. We go on to identify measurements that are positively and negatively correlated with each other, which can be used to construct a performance score for the corresponding organ systems with a minimum of redundant variables, irrelevant variables, and untested assumptions about the data. This in turn sets the stage for choosing variables from which the chronological age of an animal can be estimated. An animal whose actual age is greater than its estimated age can be interpreted as being healthier for its age an animal whose actual age is lower than its estimated age. We present several such sets of candidate variables. The software portion of the Healthspan database is freely available from the first author under the GNU Public License v2. Keywords: aging, healthspan, functional assessment, animal studies of aging, longevity, bioinformatics, variable selection, physiology of aging.

# Sub article: Sleep fragmentation and health span

**Published:** 2012-12-31

**Authors:** Keith Maslin, Stephen Treaster, Lauren Sloane, Vanessa Soto, Kathleen Fischer, Steven Austad

**Affiliations:** 1 Barshop Institute for Longevity and Aging Studies; 2 Department of Physiology; 3 Department of Cellular and Structural Biology; 4 Department of Molecular Medicine, The University of Texas Health Science Center at San Antonio, San Antonio, TX, USA; 5 State University of New York, Delhi, NY, USA

## Abstract

Sleep fragmentation is associated with aging in human populations. As part of a larger study designed to find robust, reproducible assays of health span, we used a sleep phenotyping method developed by Pack et al. (2006) to assess age-related changes in sleep patterns. Using EEG and video monitoring, Pack et al. (2006) developed and validated a simple an operational definition of sleep as a bout of /inactivity lasting ≥40s. Using their method, we developed a sleep fragmentation index by measuring the number of bouts of sleep per hour of sleep (=sleep fragmentation index) during the light and dark phases over a 24-hour period. We then used this technique to measure sleep fragmentation in 4, 20, 28, and 32-month-old male and female C57BL/6 mice to explore sex, age-related changes in sleep and sleep disruption. In combination with other assays, age-related changes in sleep patterns may offer a relatively simple, non-invasive tool for assessing healthspan in aging mice.

# Sub article: Systemic effects of decreased neuronal mTOR signaling

**Published:** 2012-12-31

**Authors:** Stacy A. Hussong, Jonathan J. Halloran, Raquel R. Burbank, Ai-Ling Lin, Vanessa Y. Soto, Veronica Galvan

**Affiliations:** 1 Department of Physiology; 2 Barshop Institute for Longevity and Aging Studies; 3 Department of Cellular and Structural Biology; 4 Research Imaging Center at the University of Texas Health Science Center at San Antonio, San Antonio, TX, USA

## Abstract

Reduction of target of rapamycin (TOR) signaling has been shown to extend lifespan in invertebrates as well as in adult mice. In other genetic models of longevity in invertebrates and mice, specific manipulations in the nervous system are sufficient to extend lifespan. To determine whether the reduction of mammalian TOR (mTOR) signaling in mature neurons of adult mice is sufficient to extend lifespan and improve health span we inducibly knocked out The mTOR complex 1 specific protein, Raptor, in adult mouse neurons after brain development was complete (2.5 months). Cre-mediated recombination of genomic DNA was detected in brain, but not in liver, and Raptor protein levels were significantly reduced after induction of Cre expression. To determine whether decreasing Raptor in neurons affected health span, we measured body mass composition, metabolism, motor coordination, muscle strength, and brain metabolite concentrations. While no significant differences in motor coordination, strength or body weight were observed among experimental groups, genetic reduction of Raptor in neurons of adult mice induced significant changes in body composition, with neuronal Raptor knock-out males becoming significantly leaner than non-transgenic controls. Adult neuronal Raptor, conditional knock-outs also showed increased levels of neuronal N-acetylaspartate, a marker of neuronal health and function. Future experiments will determine if decreased mTOR complex I signaling in adult mouse neurons is sufficient to extend lifespan and improve health span. Included in the evaluation of health span will be measures of neurological function as determined by electrophysiological and behavioral experiments.

# Sub article: Assessing frailty in mice

**Published:** 2012-12-31

**Authors:** Kathleen Fischer, Jon Gelfond, Alex Bokov, Vanessa Soto, Lauren Sloane, Keith Maslin, Stephen Treaster, Vivian Diaz, Sara Espinoza, Arlan Richardson, Steven Austad

**Affiliations:** 1 Barshop Institute; 2 Department of Physiology; 3 Department of Cellular & Structural Biology; 4 Department of Molecular Medicine; 5 Department Biostatistics & Epidemiology; 6 Department of Medicine, Division of Geriatrics, University of Texas Health Science Center at San Antonio, San Antonio, TX, USA; 7 Geriatric Research, Education and Clinical Center (GRECC), South Texas Veterans Health Care System, San Antonio, TX, USA; 8 State University of New York, Delhi, NY, USA

## Abstract

While frailty has long been recognized by physicians in the clinical setting, only recently has effort been made to standardize and quantify definitions of frailty. Fried et al. 2001 and others have used multiple measures in the hopes of developing an easily used index to evaluate age-related risks of morbidity and mortality. Among the most commonly used measures are activity, walking speed, involuntary weight loss and strength (grip strength). In order to assess age-related frailty, as opposed to ill-health more generally, two conditions should be met: firstly, the traits measured should change with age; secondly, the traits should have predictive value for increased risk of morbidity and mortality. Here we assess a combination of several potential measures of frailty in mice, including motor function (e.g. walking speed), activity (e.g. spontaneous activity), strength (e.g. grip strength), body composition and caloric expenditure (e.g. resting metabolic rate) to determine whether age-related morbidity and mortality in C57BL/6 mice can be predicted using a multivariate analysis to produce a relatively non-invasive measure of health similar to the frailty index used with humans.

# Sub article: Effects of chronic rapamycin treatment on age-related changes in cognition and motor function in genetically heterogeneous mice

**Published:** 2012-12-31

**Authors:** Rashmi Singh, Vanessa Martinez, Jonathan Halloran, Raquel Burbank, Vivian Diaz, Veronica Galvan, Elizabeth Fernandez, Randy Strong

**Affiliations:** 1 Department of Pharmacology; 2 Physiology; 3 Cellular & Structural Biology; 4 Barshop Institute for Longevity and Aging Studies, University of Texas Health Science Center, San Antonio; 5 Geriatric Research, Education and Clinical Center, South Texas Veterans Health Care System, San Antonio, TX, USA

## Abstract

It has been reported that dietary supplementation of male and female genetically heterogeneous (UM-HET3) mice with rapamycin increased median and maximum lifespan suggesting that it slows aging (Harrison et al., 2009; Miller et al., 2011). Therefore, we hypothesized that if rapamycin treatment slows aging it should also prevent or delay age-related deficits that have previously been reported in cognition and motor performance in UM-HET3 mice (Sumien et al., 2006). To test this hypothesis, we have used male and female CB6F1×C3D2F1 (UM-HET3) mice. Three groups of (N = 26 to 50) were tested: young control (4 months old), old control (24 months old) and old mice treated with rapamycin in the diet started from 12 months of age. We administered a battery of behavioral tests. Our results showed that age-related decline in locomotor and rearing activity was attenuated by rapamycin treatment in both the genders. Rapamycin treatment also attenuated the age-related decline in rotarod performance in both the genders. In addition, rapamycin treatment improves the swimming speeds of males in morris water maze test. However, we did not found any effect of rapamycin on age-related decline in grip strength. Interestingly, rapamycin improves the age-related decline in recognition memory in males. To measure anxiety and motivation, we employed the elevated plus maze and tail suspension tests respectively. No change was observed with age and treatment on anxiety and stress levels in males. However, in females rapamycin reduced the basal anxiety levels and depressive-like behavior. Altogether, our findings reveal that the increase in lifespan resulting from rapamycin supplementation is accompanied by improvements in age-sensitive behavioral traits. This study was supported by the National Institute on Aging at the National Institutes of Health (U01-AG022307).
